# Breastfeeding practices and associations with pregnancy, maternal and infant characteristics in Australia: a cross-sectional study

**DOI:** 10.1186/s13006-023-00545-5

**Published:** 2023-01-19

**Authors:** Renee Reynolds, Melanie Kingsland, Justine Daly, Milly Licata, Belinda Tully, Emma Doherty, Eva Farragher, Clare Desmet, Christophe Lecathelinais, Julianne McKie, Melanie Williams, John Wiggers, Jenna Hollis

**Affiliations:** 1 Population Health, Hunter New England Local Health District, Locked Bag 10, Wallsend, NSW 2287 Australia; 2 School of Medicine and Public Health, College of Health, Medicine and Wellbeing, The University of Newcastle, Callaghan, NSW Australia; 3 Hunter Medical Research Institute, New Lambton Heights, NSW 2305 Australia; 4 Gomeroi Nation, NSW Australia; 5 Maternity Services, Hunter New England Local Health District, New Lambton Heights, NSW Australia; 6 Maternity Services, Hunter New England Local Health District, Armidale, NSW Australia

**Keywords:** Breastfeeding cessation, Exclusive breastfeeding, Australia, Postnatal period, Determinants

## Abstract

**Background:**

Exclusive breastfeeding to six months of age is a major global public health priority. Several characteristics are known to be associated with early cessation of breastfeeding, however, limited evidence exists regarding whether women’s reported reasons for cessation are associated with maternal, pregnancy and infant characteristics. The aims of this study were to: i) describe women’s reported intention to breastfeed and their subsequent breastfeeding practices; ii) describe women’s reported reasons for breastfeeding cessation prior to the infant being five months of age; and iii) examine associations between these factors and maternal, pregnancy and infant characteristics.

**Methods:**

Telephone and online surveys were conducted between October 2019 and April 2020 with 536 women who had given birth in the previous eight to 21 weeks at four public maternity services in Australia.

**Results:**

The majority of women intended to (94%), and did, initiate (95%) breastfeeding. At the time the survey was conducted, 57% of women were exclusively breastfeeding. Women who: had less than University level education, had a pre-pregnancy BMI in the overweight or obese category, and who smoked tobacco at the time of the survey had lower odds of exclusively breastfeeding. The most common self-reported reasons for breastfeeding cessation were breastfeeding challenges (47%) and low milk supply (40%). Women aged 26–35 years and 36 + years had greater odds of reporting breastfeeding cessation due to low milk supply (OR = 2.92, 95% CI: 1.11, 7.66; OR = 5.57, 95% CI: 1.70, 18.29) compared to women aged 18–25 years. While women who had completed a TAFE certificate or diploma had lower odds of reporting this as a reason for breastfeeding cessation (OR = 0.28; 95% CI: 0.11, 0.73) compared to women who had University level education. There were no other significant associations found between characteristics and reasons for ceasing breastfeeding.

**Conclusions:**

The most common reasons for breastfeeding cessation may be modifiable through the provision of breastfeeding support in the early postpartum period, with such support being tailored to women’s age and level of education. Such support should aim to increase women's self-efficacy in breastfeeding, and be provided from the antenatal period and throughout the first six months postpartum.

**Supplementary Information:**

The online version contains supplementary material available at 10.1186/s13006-023-00545-5.

## Background

The World Health Organisation and national health authorities, including in Australia, the United States (U.S.), and the United Kingdom (U.K.), recommend that infants are exclusively breastfed for the first six months of life with breastfeeding to continue alongside the introduction of age and stage appropriate complementary foods, from 6 months of age, up to 2 years and beyond [1–4]. Despite these recommendations, global exclusive breastfeeding rates to six months of age are suboptimal in low income countries (51.2%), low-middle income countries (46.7%) and upper-middle income countries (37.0%) [5]. Exclusive breastfeeding rates to six months of age are also suboptimal in many high income countries including Australia (15.4%), the U.S. (25.6%) and the U.K. (1%) [5–9].

Intention to breastfeed, reported in the prenatal period, is a strong predictor of breastfeeding initiation and is associated with longer duration of breastfeeding [10]. The majority of Australian women (96%) initiate breastfeeding [11] however rates of exclusive breastfeeding dramatically decline in the following months with only 39% of infants being exclusively breastfed by aged three months [1, 2, 11, 12]. While intention and initiation of breastfeeding is high among women at a population level, some maternal characteristics have been found to be associated with decreased intention, initiation and duration of breastfeeding. A 2014 systematic review of 19 mostly prospective cohort studies found that maternal obesity is associated with decreased intention, initiation and duration of breastfeeding [13]. An additional 2016 cross-sectional study of 229 women in the U.S. found that while women with high risk obstetric conditions such as gestational and pre-gestational diabetes mellitus, substance abuse and pre-eclampsia had a high breastfeeding intention rate, this did not translate into high initiation rates [14].

In Australia, the Australian National Infant Feeding Survey (*N* = 28,759) and other large observational studies (range: *N* = 889–17,564) have similarly found that early cessation of breastfeeding (prior to six months of age) is associated with a number of maternal characteristics, including younger age (less than 25 years old), lower socioeconomic status (SES), lower education levels, daily cigarette smoking, caesarean or assisted vaginal birth, intimate partner violence and lack of social/partner support [11, 15–19]. The National Survey also found that the rate of decline of exclusive breastfeeding is greater for women with Aboriginal infants than those with non-Aboriginal infants, with 7% of Aboriginal infants exclusively breastfed to six months of age compared to 16% of non-Aboriginal infants [11]. A recent systematic review also found that Aboriginal women initiate and maintain breastfeeding at lower rates than non- Aboriginal women [20].

There is limited evidence that comprehensively examines associations between maternal and pregnancy characteristics and women’s reported reasons for breastfeeding cessation prior to six months of age [21–23]. The largest and most recent of these studies, conducted in the U.S. with 7,837 women enrolled in a peer counselling breastfeeding program, found that maternal age, race and ethnicity, marital status and newborn outcomes were differentially associated with specific reasons for ceasing breastfeeding such as women’s preference, women’s/infants medical conditions, breastfeeding challenges [21]. A longitudinal study [24] and two cross-sectional studies [15, 17] conducted in Australia with samples ranging from 209 – 2,669 women found the most common reported reason for ceasing exclusive breastfeeding or breastfeeding altogether prior to six months of age was perceived insufficient milk supply. [15, 24]. The next most common reasons for cessation were issues around the infant not being satisfied from breast milk and trouble latching on [15, 24].

While these studies provide important insight into the specific reasons why women cease infant breastfeeding before the infant is six months of age, no Australian studies have investigated associations between women’s reasons for early breastfeeding cessation and maternal, pregnancy and infant characteristics, such as maternal and infant age, identifying as Aboriginal, level of education, marital status, pregnancy risk level and lifestyle behaviours postpartum. Understanding of such relationships may assist in developing public health initiatives to provide tailored, and timely support to women and their families as part of routine antenatal and postnatal care to increase rates of exclusive breastfeeding [25]. This is of particular importance for specific groups of women, including Aboriginal women, younger women and women with high risk pregnancies who appear more likely to cease breastfeeding before infants are 6 months of age.

As no Australian study has comprehensively examined breastfeeding intention, practices and women’s reported reasons for early cessation, and their association with maternal, pregnancy and infant characteristics within the same cohort, a study was undertaken to i) describe women’s reported intention to breastfeed and their subsequent breastfeeding practices; ii) describe women’s reported reasons for breastfeeding cessation prior to the infant being five months of age; and iii) examine associations between these factors and maternal, pregnancy and infant characteristics.

## Methods

This study is reported in accordance with the Strengthening the Reporting of Observational studies in Epidemiology guidelines [26].

### Study design and setting

A cross-sectional telephone/online survey was conducted between October 2019 and April 2020 with women who had recently given birth in the Hunter New England Local Health District, New South Wales (NSW), Australia.

### Sample and recruitment

#### Participants

Women who had participated in a previous study during their pregnancy were invited to participate. Details of the previous study's methods are described elsewhere [27]. Women were eligible for inclusion in this postnatal survey if they: had consented to be followed up after birth in the previous study, were 18 years of age or older, had given birth between 8 and 21 weeks prior, had a level of English proficiency that enabled them to undertake the survey unaided and had not had an adverse pregnancy related outcome, including stillbirth and miscarriage. The survey's primary purpose was to assess women’s recollection of care received in the first 5 months following birth.

#### Recruitment procedure

Each week during the study period, 60 women from the eligible sample were mailed an information statement outlining the purpose of the study and inviting them to participate. The information statement included a toll free or freephone number that women could call to decline participation in the survey.

One week after the information statement was mailed, non-Aboriginal women were contacted via telephone and invited to participate in a Computer Assisted Telephone Interview (CATI). Women who declined to participate when called were offered the opportunity to complete the survey online.

As per advice received through cultural consultation, Aboriginal women were sent a text message following the mail out of the information statement, offering a choice to complete the survey via telephone, online or to opt out of participation. Aboriginal women who opted to complete the survey online were sent a link to the survey which was active for two weeks. For women who did not reply to the text message after three days, attempts were made to contact via telephone and invite them to participate in the survey.

Attempts to contact women were conducted over a two week period with up to 10 contact attempts made. Women could decline participation at any point during the CATI or online survey.

### Data collection procedures

Both the telephone and online surveys were developed using REDCap (Research Electronic Data Capture) electronic data capture tools [28, 29]. The telephone survey was conducted by trained and experienced female interviewers and the survey was pilot tested before use. Aboriginal women were given the choice of undertaking the survey with an Aboriginal interviewer. Additional data was obtained directly from women’s electronic medical records and linked to individual survey responses.

### Measures

#### Women’s demographic and pregnancy characteristics

Women’s residential postcode, age, pregnancy risk level, pregnancy outcome and the infants' date of birth were obtained via the woman’s electronic medical record. Women reported the following characteristics as part of the initial survey during pregnancy: marital status, highest level of education, whether this was their first pregnancy, and smoking status and alcohol consumption during pregnancy (using the validated Alcohol Use Disorders Identification Test Consumption (AUDIT-C) tool [30, 31]). The following characteristics of women and their most recent pregnancy were reported as part of this postnatal survey: Aboriginal and/or Torres Strait Islander origin, single or multiple pregnancy, gestational diabetes, pre-eclampsia, caesarean section, height, pre-pregnancy weight, current smoking status and current alcohol consumption (using the AUDIT-C).

#### Women’s intention to breastfeed and breastfeeding practices

Women were asked ‘Did you plan to breastfeed your baby?’ (yes; no; don’t know), followed by ‘Are you currently breastfeeding your baby?’ (yes – exclusively; yes – breastfeeding and formula feeding; yes – breastfeeding and commenced solid foods; no; don’t know). The following definition of exclusive breastfeeding was provided to women who asked for clarity on the definition of exclusive breastfeeding: ‘Exclusively breastfeeding means that the infant receives only breast milk. No other liquids or solids are given – not even water—with the exception of oral rehydration solution, or drops/syrups of vitamins, minerals or medicines.’ [32]. Women who indicated that they were not currently breastfeeding were asked ‘Did you try to breastfeed or breastfeed for a while?’ (yes; no; don’t know). Women who did try to breastfeed or breastfed for a while were asked ‘How long did you breastfeed for?’ (less than one month; one to two months; three or more months; don’t know).

#### Reasons for not initiating breastfeeding or breastfeeding cessation

To assess women’s reasons for not initiating breastfeeding, women were asked in an open-ended question: ‘We know there are many reasons for not breastfeeding. Why did you decide not to?’ Women who initiated breastfeeding but had ceased breastfeeding prior to participating in the survey were asked in an open ended question ‘We know there are many reasons for moving on from breastfeeding. Why did you move on?’. Responses were not prompted and women could nominate multiple reasons.

### Analysis

Data analysis was conducted using SAS 9.3 (SAS Institute, Cary, NC) [33]. As per Table 2 condensed categories were created for maternal age, Aboriginal and Torres Strait Islander origin, highest level of education completed and reasons for breastfeeding cessation. Responses of 'don't know' were classified as 'no'. Categorisation of reasons for breastfeeding cessation were based on a similar study conducted by Rozga et al.,2015 [21]. The types of reasons in each category are listed in Table 1. For the purpose of assessing associations between maternal and pregnancy characteristics, age of the infant and exclusive breastfeeding at the time of survey completion, categories for the question ‘Are you currently breastfeeding your baby?’ were condensed to ‘Yes, exclusively’ and ‘No, not exclusively’ (including the response options of ‘yes – breastfeeding and formula feeding’, ‘yes – breastfeeding and commenced solid foods’ and ‘no’). Women’s height and pre-pregnancy weight was used to calculate pre-pregnancy body mass index (BMI) using the formula BMI = kg/m^2^. BMI was categorised at underweight (< 18.5 kg/m^2^), healthy weight (18.5–24.9 kg/m^2^), overweight (25.0–29.9 kg/m^2^) or obese (≥ 30 kg/m^2^). Model of antenatal care was used to indicate pregnancy risk level. Low risk was defined as hospital and community-based midwifery clinics, midwifery group practice continuity of care and multidisciplinary care for women with social vulnerabilities. High risk was defined as specialist medical clinics and multi-disciplinary care for women with complex medical needs. Aboriginal Maternal Infant Health Services (AMIHS) provide culturally appropriate maternity care to Aboriginal women for both low and high risk pregnancies. As such, women who were identified as receiving their antenatal care through an AMIHS were excluded from the pregnancy risk level analyses.

**Table 1:** Categorisation of survey responses for breastfeeding cessation

Category	Responses
Perceived insufficient milk supply	Breast milk alone did not satisfy my baby I thought that my baby was not gaining enough weight I didn’t have enough milk Baby just didn’t settle on breastmilk
Breastfeeding challenges	I had trouble getting the milk flow to start My baby had trouble sucking or latching on Breastfeeding was too painful Baby was too difficult Baby began biting My baby lost interest or began to wean him or herself
Woman's preference	Breastfeeding was too tiring Breastfeeding was too inconvenient I wanted or needed someone else to feed my baby I wanted to smoke again/I was smoking I had other kids at home I didn’t feel comfortable breastfeeding in public Anxious from a previous experience
Medical conditions	I had an infection (mastitis) I was unwell or taking medications Medical advice Baby in the neonatal intensive care unit or unwell Baby has allergies Baby was tongue tied Baby suffers from reflux
Other	Oestrogen sensitivity Partner left Surrogate pregnancy

The risk of harm categories used for analysing alcohol consumption risk level are consistent with Australian national guidelines for pregnancy and have been applied for breastfeeding given the same guideline recommendation to not consume alcohol: ‘no risk of harm’ for an AUDIT-C score of 0, ‘low risk of harm’ for an AUDIT-C score of 1–2, ‘medium risk of harm’ for an AUDIT-C score of 3–4 and a ‘high risk of harm’ AUDIT-C score of 5 and above [34]. Women whose postcodes were ranked in the top 50% of NSW postcodes, based on the Australian Bureau of Statistics 2016 Socio-Economic Indexes For Australia [35], were categorised as ‘least disadvantaged’, while those in the lower 50% were categorised as ‘most disadvantaged’.

Descriptive statistics were used to describe maternal and pregnancy characteristics; the infants age at survey completion; women’s plans to breastfeed; breastfeeding initiation; current breastfeeding practice and reasons for not initiating breastfeeding. Simple logistic regression models were used to identify associations between each characteristic and the following outcomes: intention to breastfeed (13 models), breastfeeding initiation (13 models), and reasons for breastfeeding cessation (14 models). Additionally, simple and multivariable logistic regression models (15 models) were used to identify associations between all characteristics and whether women were exclusively breastfeeding at the time of survey completion. The ‘other’ category for breastfeeding cessation was excluded from logistic regression analyses due to a low response rate. An alpha level of 0.05 was set to denote statistical significance. Due to the exploratory nature of the study no further adjustment was made to the alpha level for multiple testing.

Assuming a sample of 500 women, 80% power and a significance level of *p* < 0.05, allowed a detection of 12.4% significant difference between characteristics for each dichotomous outcome.

## Results

Over the seven month study period, a total of 1032 women were invited to participate. Of these women, 875 (85%) were able to be contacted and 871 (84%) were eligible to complete the survey. Of the women who were eligible, 566 (65%) consented to participate, and a total of 536 (62%) completed the survey. Women had significantly greater odds of participating in the survey if they had completed University level education compared to women who had completed high school or less (OR = 2.06; 95% CI: 1.46, 2.92) and women who had completed a TAFE certificate or diploma (OR = 1.61; 95% CI: 1.17, 2.24).

Characteristics of the women who participated in the survey such as, demographics, age of infant, parity, method of birth, pregnancy risk level and health behaviours are described in Table 2.

**Table 2:** Maternal and pregnancy characteristics of participants and age of infant

Characteristic (*N* = 536)	n (%) or mean (SD)
Age of woman, Mean (SD)	30.75 (4.97)
18—25 years	81 (15.1%)
26—35 years	364 (67.9%)
36 + years	91 (17%)
Age of infant, Mean (SD)	3.26 (0.18)
2 months	103 (19.2%)
3 months	213 (39.7%)
4 months	199 (37.1%)
5 months	21 (3.9%)
Woman identifies as Aboriginal or Torres Strait Islander, or both	27 (5.0%)
Marital status	
Never married	43 (8.0%)
Married or de facto	483 (90.1%)
Separated/divorced	10 (1.9%)
Highest level of education	
Completed high school or less	122 (22.8%)
Completed TAFE Certificate or Diploma	176 (32.8%)
Completed University, CAE, Degree or higher	238 (44.4%)
Index of disadvantage	
Most disadvantaged	288 (53.7%)
Least disadvantaged	248 (46.3%)
Pre-pregnancy BMI (*N* = 484)	
Underweight (< 18.5 kg/m^2^)	14 (3.0%)
Healthy weight (18.5–24.9 kg/m^2^)	228 (47.1%)
Overweight (25.0–29.9 kg/m^2^)	111 (22.9%)
Obese (≥ 30.0 kg/m^2^)	131 (27.0%)
First pregnancy	187 (34.9%)
Caesarean birth	159 (29.7%)
Pre-eclampsia	10 (1.9%)
Gestational diabetes	56 (10.5%)
Pregnancy risk level (*N* = 533)	
Low risk	369 (69.2%)
High risk	164 (30.8%)
Smoked tobacco during pregnancy	40 (7.5%)
Alcohol consumption during pregnancy ^a^	
No risk of harm (score = 0)	477 (89.0%)
Low risk of harm (score = 1–2)	58 (10.8%)
Medium risk of harm (score = 3–4)	1 (0.2%)
High risk of harm (score = 5 +)	0 (0%)
Smoked tobacco at time of survey	34 (6.3%)
Alcohol consumption at time of survey ^a^	
No risk of harm (score = 0)	228 (42.5%)
Low risk of harm (score = 1–2)	206 (38.4%)
Medium risk of harm (score = 3–4)	82 (15.3%)
High risk of harm (score = 5 +)	20 (3.3%)

^a^Measured using AUDIT-C

### Women’s intention to breastfeed and breastfeeding initiation, and association with maternal, pregnancy and infant characteristics

Almost all women (*n* = 503; 94%) reported that they had planned to breastfeed their infant, and 99% (*n* = 498) of these women did initiate breastfeeding. Of the women who did not plan to breastfeed their infant (*n* = 33), 39% (*n* = 13) did initiate breastfeeding after birth. In total, 511 (95%) women reported that they initiated breastfeeding and 25 (5%) women did not initiate any breastfeeding.

Table 3 shows the results of simple logistic regression assessing associations between women’s intention to breastfeed and breastfeeding initiation and maternal, pregnancy and infant characteristics at the time of survey completion. Of the 13 characteristics, three were significantly associated with both intention to breastfeed and initiation of breastfeeding. Women who had never married had lower odds of planning to breastfeed their infant (OR = 0.29; 95% CI: 0.11, 0.86) and initiating breastfeeding following birth (OR = 0.25; 95% CI: 0.10, 0.67) than women who were married/in a de facto relationship. Women who had completed high school or less, or had completed a TAFE certificate or diploma, had lower odds of planning to breastfeed their infant compared to women who had completed University level education (OR = 0.15; 95% CI: 0.05, 0.43; OR = 0.27; 95% CI: 0.09, 0.77). Women who had completed high school or less had lower odds of initiating breastfeeding than women who had completed University level education (OR = 0.16; 95% CI: 0.05, 0.50). Women who smoked during pregnancy had lower odds of planning to breastfeed (OR = 0.26; 95% CI: 0.11, 0.65) and initiating breastfeeding (OR = 0.29; 95% CI: 0.10, 0.83) than women who did not smoke during pregnancy.

**Table 3:** Associations between participants’ characteristics and breastfeeding intention and initiation

Characteristic^a^	Planned to breastfeed (*N* = 503)			Initiated breastfeeding (*N* = 511)		
	**n (%)**	**Odds ratio (95% CI)**	***p*-value**	**n (%)**	**Odds ratio (95% CI)**	***p*-value**
**Age of woman (years)**			.29			.44
18-25^b^	73 (90.1%)	1.0		346 (95.1%)	1.0	
26–35	343 (94.2%)	1.79 (0.76, 4.20)		89 (97.8%)	1.26 (0.46, 3.51)	
36 +	87 (95.6%)	2.38 (0.69, 8.24)		76 (93.8%)	2.93 (0.55, 15.52)	
**Aboriginal or Torres Strait Islander, or both**			.07			.12
Yes	23 (85.2%)	0.35 (0.11, 1.07)		24 (88.9%)	0.36 (0.10, 1.29)	
No^b^	480 (94.3%)	1.0		487 (95.7%)	1.0	
**Marital status**			.033^c^			.026^c^
Never married	36 (83.7%)	0.29 (0.11, 0.86)		37 (86.1%)	0.25 (0.09, 0.82)	
Separated or divorced	10 (100%)	0.78 (0.15, ∞)		10 (100%)	0.56 (0.11, ∞)	
Married or de facto^b^	457 (94.6%)	1.0		464 (96.1%)	1.0	
**Education**			.002^c^			.006^c^
Completed high school or less	107 (87.7%)	0.15 (0.05, 0.43)		110 (90.2%)	0.16 (0.05, 0.50)	
Completed TAFE Certificate or Diploma	163 (92.6%)	0.27 (0.09, 0.77)		167 (94.9%)	0.32 (0.10, 1.05)	
Completed University, CAE, Degree or higher^b^	233 (97.9%)	1.0		234 (98.3%)	1.0	
**Disadvantage**			.65			.52
Most disadvantaged	269 (93.4%)	0.85 (0.42, 1.73)		273 (94.8%)	0.76 (0.34, 1.73)	
Least disadvantaged^b^	234 (94.4%)	1.0		238 (96.0%)	1.0	
**Pre-pregnancy BMI**	**(*N* = 459)**		.23	**(*N* = 464)**		.24
Underweight (< 18.5 kg/m^2^)	14 (100%)	0.96 (0.19, ∞)		13 (92.9%)	0.47 (0.05, 4.07)	
Healthy weight^b^ (18.5–24.9 kg/m^2^)	217 (95.2%)	1.0		220 (96.5%)	1.0	
Overweight (25.0–29.9 kg/m^2^)	108 (97.3%)	1.82 (0.47, 10.38)		109 (98.2%)	1.98 (0.41, 9.48)	
Obese (≥ 30.0 kg/m^2^)	120 (91.6%)	0.55 (0.21, 1.46)		122 (93.1%)	0.49 (0.19, 1.31)	
**Pregnancy**			.19			.052
First pregnancy	179 (95.7%)	1.73 (0.76, 3.91)		183 (97.9%)	2.93 (0.99, 8.66)	
Subsequent pregnancy^b^	324 (92.8%)	1.0		328 (94.0%)	1.0	
**Type of birth**			.93			.11
Vaginal	354 (93.9%)	1.03 (0.48, 2.22)		363 (96.3%)	1.93 (0.86, 4.34)	
Caesarean^b^	149 (93.71)	1.0		148 (93.1%)	1.0	
**Pre-eclampsia**			.61			1.00
No	494 (93.9%)	1.72 (0.21, 13.96)		501 (95.3%)	1.46 (0.00, 7.37)	
Yes^b^	9 (90.0%)	1.0		10 (100%)	1.0	
**Gestational Diabetes**			.75			.36
No^b^	451 (94.0%)	1.0		459 (95.6%)	1.0	
Yes	52 (92.9%)	0.84 (0.28, 2.47)		52 (92.9%)	0.59 (0.20, 1.80)	
**Pregnancy risk level**	**(*N* = 501)**		.40	**(*N* = 508)**		.56
Low risk	349 (94.6%)	1.38 (0.66, 2.89)		353 (95.7%)	1.28 (0.55, 2.96)	
High risk^b^	152 (92.7%)	1.0		155 (94.5%)	1.0	
**Smoked tobacco during pregnancy**			.004^c^			.021^c^
No^a^	470 (94.8%)	1.0		476 (96.0%)	1.0	
Yes	33 (82.5%)	0.26 (0.11 – 0.65)		35 (87.5%)	0.29 (0.10, 0.83)	
**Alcohol consumption during pregnancy (AUDIT-C)**			.29			.53
No risk of harm (score = 0)^b^	445 (93.3%)	1.0		453 (95%)	1.0	
Low risk of harm (score = 1–2)	57 (98.3%)	4.09 (0.66 – 169.78)		57 (98.3%)	3.02 (0.47, 126.33)	
Medium risk of harm (score = 3–4)	1 (100%)	0.07 (0.00, ∞)		1 (100%)	0.05 (0.00, ∞)	

^a^Age of infant not included as it is not relevant to breastfeeding intention or initiation

^b^Reference value for Odds Ratio

^c^Statistically significant at (α = 0.05)

### Breastfeeding at time of survey and associations with maternal, pregnancy and infant characteristics

**Fig. 1:**
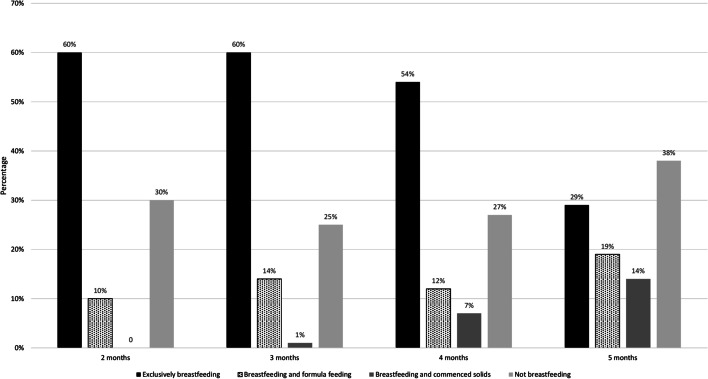
Breastfeeding practices by age of the infant at time of survey completion

At the time of the survey, 390 (73%) women were currently breastfeeding their infant. Specifically, 57% (*n* = 304) reported breastfeeding exclusively, 13% (*n* = 68) were breastfeeding and formula feeding and 3% (*n* = 18) were breastfeeding and had commenced solids. Figure 1 depicts current breastfeeding practices at the time of survey completion by the age of the infant.

Of the women who had initiated breastfeeding but who were not currently breastfeeding their infant at the time of survey completion (*n* = 121), 51% (*n* = 62) had breastfed for less than one month, 35% (*n* = 42) had breastfed for one to two months and 14% (*n* = 14) had breastfed for three or more months.

Table 4 shows the results of analysis assessing associations between maternal and pregnancy characteristics and babies' age and whether they were exclusively breastfeeding at the time of survey completion. Adjusting for all characteristics: women who had completed high school or less or a TAFE certificate/diploma (OR = 0.40; 95% CI: 0.22, 0.72; OR = 0.46; 95% CI: 0.28, 0.76), women who had a pre-pregnancy BMI in the overweight or obese range (OR = 0.50; 95% CI: 0.30, 0.86; OR = 0.30; 95% CI: 0.18, 0.50) and women who smoked tobacco at the time of the survey (OR = 0.09; 95% CI: 0.02, 0.33) had lower odds of exclusively breastfeeding at time of survey completion. Simple logistic regression models found additional characteristics associated with lower odds of exclusively breastfeeding at the time of survey as seen in Table 4.

**Table 4:** Associations between characteristics and exclusive breastfeeding at time of survey completion

Characteristic	Exclusively breastfeeding (*N* = 304) n (%)	Unadjusted Odds ratio (95%CI)	*p*-value	Adjusted Odds Ratio (95% CI)	*p*-value
**Age of woman (years)**			.004^c^		.72
18-25^b^	33 (40.7%)	1.0		1.0	
26–35	222 (61.0%)	2.27 (1.39, 3.71)		1.19 (0.62, 2.29)	
36 +	49 (53.9%)	1.70 (0.93, 3.11)		0.98 (0.42, 2.25)	
**Age of infant (months)**			.046^c^		.11
2 months^b^	62 (60.2%)	1.0		1.0	
3 months	128 (60.1%)	1.00 (0.62, 1.61)		1.24 (0.70, 2.20)	
4 months	108 (54.3%)	0.78 (0.48, 1.27)		0.84 (0.47, 1.51)	
5 months	6 (28.6%)	0.26 (0.09, 0.74)		0.36 (0.11, 1.15)	
**Aboriginal or Torres Strait Islander, or both**			.19		.51
Yes	12 (44.4%)	0.59 (0.27, 1.30)		1.37 (0.54, 3.51)	
No^b^	292 (57.4%)	1.0		1.0	
**Marital status**			.027^c^		.90
Never married	16 (37.2%)	0.42 (0.22, 0.80)		0.95 (0.40, 2.29)	
Separated or divorced	5 (50.0%)	0.71 (0.20, 2.47)		1.38 (0.32, 5.87)	
Married or de facto^b^	283 (58.6%)	1.0		1.0	
**Education**			< .001^c^		.002^c^
Completed high school or less	51 (41.8%)	0.28 (0.18, 0.44)		0.40 (0.22, 0.72)	
Completed TAFE Certificate or Diploma	82 (46.6%)	0.34 (0.23, 0.51)		0.46 (0.28, 0.76)	
Completed University, CAE, Degree or higher^b^	171 (71.9%)	1.0		1.0	
**Disadvantage**			.020^c^		.053
Most disadvantaged	150 (52.1%)	0.66 (0.47, 0.94)		0.66 (0.43, 1.00)	
Least disadvantaged^b^	154 (62.1%)	1.0		1.0	
**Pre-Pregnancy BMI**	**(*N* = 280)**		< .001^c^		< .001^c^
Underweight (< 18.5 kg/m^2^)	10 (71.4%)	1.11 (0.34, 3.65)		1.34 (0.35, 5.16)	
Healthy weight^b^ (18.5–24.9 kg/m^2^)	158 (69.3%)	1.0		1.0	
Overweight (25.0–29.9 kg/m^2^)	59 (53.2%)	0.50 (0.32, 0.80)		0.50 (0.30, 0.86)	
Obese (≥ 30.0 kg/m^2^)	53 (40.5%)	0.30 (0.19, 0.47)		0.30 (0.18, 0.50)	
**Pregnancy**			.86		.77
First pregnancy	107 (57.2%)	1.03 (0.72, 1.48)		0.93 (0.58, 1.49)	
Subsequent pregnancy^b^	197 (56.5%)	1.0		1.0	
**Type of birth**			.17		.73
Vaginal	221 (58.6%)	1.30 (0.89, 1.88)		1.08 (0.69, 1.70)	
Caesarean^b^	83 (52.2%)	1.0		1.0	
**Pre-eclampsia**			.10		.67
No	301 (57.2%)	3.12 (0.80, 12.20)		1.41 (0.30, 6.57)	
Yes^b^	3 (30.0%)	1.0		1.0	
**Gestational Diabetes**			.62		.53
No^b^	274 (57.1%)	1.0		1.0	
Yes	30 (53.6%)	0.87 (0.50, 1.51)		1.24 (0.63, 2.47)	
**Pregnancy risk level**	**(*N* = 303)**		< .001^c^		.10
Low risk	229 (62.1%)	1.99 (1.37, 2.89)		1.46 (0.92, 3.32)	
High risk^b^	74 (45.1%)	1.0		1.0	
**Smoked tobacco at time of survey**			< .001^c^		< .001^c^
No^a^	301 (60.0%)	1.0		1.0	
Yes	3 (8.8%)	0.06 (0.02, 0.21)		0.09 (0.02, 0.33)	
**Alcohol consumption at time of survey**			.003^c^		.06
No risk of harm (score = 0)^a^	141 (61.8%)	1.0		1.0	
Low risk of harm (score = 1–2)	119 (57.8%)	0.84 (0.57, 1.24)		0.79 (0.50, 1.25)	
Medium risk of harm (score = 3–4)	41 (50.0%)	0.62 (0.37, 1.03)		0.49 (0.27, 0.89)	
High risk of harm (score = 5 +)	3 (15.0%)	0.11 (0.03, 0.38)		0.29 (0.07, 1.26)	

^a^ Mixed feeding refers to breastfeeding plus formula or breastfeeding plus solids

^b^ Reference value for Odds Ratio

^c^ Statistically significant at (α = 0.05)

### Reasons for not initiating breastfeeding or breastfeeding cessation and associations with maternal, pregnancy and infant characteristics

Of the 536 women surveyed, 25 women did not initiate breastfeeding. The most common responses for not breastfeeding were: '*I had a previous negative experience with breastfeeding'* (*n* = 7)*, 'I have difficulties with lactation'* (*n* = 6) and '*Breastfeeding did not appeal to me/fit with my lifestyle'* (*n* = 6).

The most commonly reported reasons for breastfeeding cessation were breastfeeding challenges (47%) and perceived insufficient milk supply (40%). Age and education were associated with ceasing breastfeeding due to perceived insufficient milk supply. Women aged 26–35 years and 36 + years (OR = 2.92, 95% CI: 1.11, 7.66; OR = 5.57, 95% CI: 1.70, 18.29) were at greater odds of reporting this as a reason for ceasing breastfeeding than women aged 18–25 years. While women who had completed a TAFE certificate or diploma had lower odds of reporting this as a reason for ceasing breastfeeding (OR = 0.28; 95% CI: 0.11, 0.73) compared to women who had completed University level education. There were no other significant associations found between characteristics and reasons for ceasing breastfeeding. Further detail on analyses between maternal, pregnancy and infant characteristics and common reasons cited for breastfeeding cessation can be seen in Additional file 1.

## Discussion

This is the first study to examine associations between maternal, pregnancy and infant characteristics and reasons for breastfeeding cessation prior to five months of age in the Australian context. Almost all women planned and initiated breastfeeding after birth. However, rates of exclusive breastfeeding declined in the following months, with only 29% of those surveyed at five months exclusively breastfeeding. The most common reason for ceasing breastfeeding was breastfeeding challenges followed by perceived insufficient milk supply. A number of associations were identified between maternal and pregnancy characteristics, and breastfeeding initiation, intention, and reasons for cessation, providing additional context regarding women's breastfeeding experiences and barriers to exclusive breastfeeding. No associations between reasons for cessation and infant age were identified.

The high proportion (95%) of women that initiated breastfeeding and were exclusively breastfeeding at two months (60%) is consistent with findings from the 2010 Australian National Infant Feeding Survey (96%, 60.3%, *N* = 28,759) [11]. Of the women surveyed at five months post birth, 29% were exclusively breastfeeding, which is similar to national breastfeeding rates (35.4% of babies being exclusively breastfed to six months of age) [36], but less than the global rate of 41% [37]. Additionally, despite global and national recommendations to introduce complementary foods from six months of age [38], infants were reported to be introduced to solids as early as three months, with 14% of breastfeeding women surveyed at the time that their infants were five months having already introduced solid foods. However it is important to note that the question relating to feeding practices at the time of the survey did not include an option for combined formula feeding and solids and so the proportion of babies introduced to solids before six months of age in this group may be much higher. These findings suggest that global and national infant feeding guidelines, including breastfeeding recommendations, are not being met for large proportions of women and the benefits of exclusive breastfeeding to six months of age are not being maximised.

The incremental decline in exclusive breastfeeding over the first few months postpartum, demonstrates the importance of the antenatal and early postpartum periods as pivotal times to provide women with support to address identified reasons for not exclusively breastfeeding. Systematic review evidence indicates that education and support-based interventions delivered until 4–6 months postpartum via telephone, text and websites are effective in extending the duration of breastfeeding practices in high-income countries [39]. In Australia, pregnant women are recommended to be offered breastfeeding support as part of routine antenatal and postnatal care [2, 40–42]. However, women internationally [43] and in Australia [44] report that early breastfeeding support is inadequate, and at times unacceptable and inappropriate. Targeted use of technology, such as a universal telephone text messaging service for women with links to support services and evidence-based educational websites, may assist in providing timely assistance and improving breastfeeding practices. Further research to determine the most effective and acceptable support (i.e. mode, antenatal/postnatal timing, type e.g. education and behavioural support) for women experiencing different breastfeeding challenges may enable tailored, timely breastfeeding support to be provided in resource-constrained settings, such as antenatal and postnatal care.

In contrast to a recent systematic review [20], there was no significant difference in the rates of initiation of breastfeeding or exclusive breastfeeding at the time of the survey between Aboriginal women compared to non-Aboriginal women. However as previously mentioned, the review found that rates of breastfeeding varied greatly between the included studies due to the lack of standard measures and definitions of breastfeeding [20]. Future breastfeeding research should seek to address this methodological limitation by using standardised measures and definitions to describe breastfeeding practices to accurately capture women’s breastfeeding experiences and enable between study comparisons. Any strategies developed to improve rates of exclusive breastfeeding should be co-developed by Aboriginal communities and partners to ensure cultural appropriateness and benefit for Aboriginal women [45]. While evidence exists on the effectiveness of a joint cultural governance approach in other areas of maternal care [46], effectiveness of developing and implementing breastfeeding interventions for Aboriginal populations in Australia and globally is under-researched [47].

Our study identified several maternal and pregnancy characteristics associated with lower breastfeeding intention and practices which provide some insight as to women who may experience greater barriers to exclusive breastfeeding and may benefit from additional or more tailored breastfeeding support. Women who had never married, had lower education levels, or smoked during pregnancy were less likely to report an intention to breastfeed and to initiate breastfeeding. Further, women with lower education levels, who had a pre-pregnancy BMI in the overweight or obese range, or who smoked at the time of the survey, were more likely to report ceasing exclusive breastfeeding prior to five months. These findings are consistent with past research [10, 15, 16, 48, 49] showing that sociodemographic characteristics and behavioural and health profiles are associated with poorer breastfeeding outcomes highlighting the importance of supporting all women to exclusively breastfeed and the need to address risks such as smoking, alcohol consumption and weight gain as part of routine antenatal care.

Perceived insufficient milk supply and breastfeeding challenges were the most commonly cited reasons for breastfeeding cessation, consistent with previous research [10, 15, 17, 24, 25, 50]. Women who were older (26 + years) had higher odds than women aged 18–25 years and women who had attained TAFE/diploma level education had lower odds compared to University educated women of reporting breastfeeding cessation due to perceived insufficient milk supply. There is limited research exploring why women with such characteristics are associated with this barrier to breastfeeding [51]. One theory is that older and University educated women may be more likely to return to employment sooner following birth, reducing their capacity to breastfeed, leading to early supplementation and consequently decreasing their milk supply [51, 52]. While insufficient milk supply is consistently reported as a common reason that women attribute to breastfeeding cessation, it is estimated that only 5% of women experience physiologic insufficient milk supply [10]. A recent systematic review of 27 observational and qualitative studies found that delayed breastfeeding initiation, lack of knowledge on exclusive breastfeeding and formula supplementation were the main contributing factors to perceived insufficient milk supply [53]. Additionally, there was a strong negative association with breastfeeding self-efficacy and a moderate negative association with infant sucking ability and planned duration of breastfeeding [53]. A systematic review of 11 studies (3 quasi-experimental design and 8 randomised controlled trials) found that interventions delivered postnatally both in the hospital and community, and informed by breastfeeding self-efficacy theory, were most efficacious in increasing breastfeeding self-efficacy and outcomes [54]. Education interventions (providing information, demonstration, and/or discussion) were more effective than support-based interventions (providing social support, counselling or consultation) in improving breastfeeding self-efficacy but this did not translate in to breastfeeding outcomes [54]. More research is needed to determine if the timing, setting and frequency of interventions impacted the success of education interventions compared with support-based interventions in increasing breastfeeding self-efficacy, and the intervention components effective in reducing breastfeeding cessation due to perceived insufficient milk supply, particularly in women who are above 26 years of age and University educated.

The most commonly reported breastfeeding challenge in this study was that the infant had trouble sucking or latching onto the breast and that breastfeeding was too painful. No associations between maternal and pregnancy characteristics and breastfeeding challenges were identified suggesting it is a common challenge experienced by women with diverse socioeconomic and health experiences. Challenges with infants latching and nipple pain are interrelated and commonly reported by breastfeeding women [55]. Providing women with anticipatory guidance on expected duration of nipple pain, and educating and correcting latching and positioning as part of antenatal and early postnatal care may assist with maintenance of early breastfeeding during this period and ultimately extend breastfeeding duration and exclusivity [56].

The results of the study should be considered within the context of a number of strengths and limitations. Firstly, the study was conducted with a large sample of women from diverse socioeconomic backgrounds, with sample demographic characteristics comparable to the Australian National Infant Feeding Survey [11]. However, women who completed University level education were more likely to participate in the survey, therefore the findings should be interpreted with such consideration. Women were sampled between 8 and 21 weeks postpartum to enhance the accuracy of women’s recall of breastfeeding practices and care received in the early postpartum period, therefore the breastfeeding practices reported may not be a true representation of breastfeeding practices up to six months of age due to the variability in age of the infant. However, as guidelines state that exclusive breastfeeding should continue up to six months of age, this data provides evidence that Australian breastfeeding rates continue to fall short of recommendations. Despite providing interviewers with a definition of exclusive breastfeeding, the statement was only read out to women who required clarification and therefore some women who reported exclusively breastfeeding may not have accurately understood this term. The results may be influenced by misreporting or social desirability bias due to the self-reported nature of the survey [57]. The study was conducted in four maternity services within one local health district in NSW, Australia, therefore generalisation of the study findings to other regions of Australia and globally is unknown, however the study prevalence rates are consistent with national data [11, 12]. As a small number of Aboriginal women completed the survey (*n* = 27), analysis of Aboriginal specific health care providers such as Aboriginal Medical Services and AMIHS was limited and should be interpreted with such consideration. While the groups examining associations between reasons for cessation and characteristics are small and should be interpreted with caution, they are appropriate for an exploratory study. Further research with a larger sample size is warranted.

## Conclusions

While the vast majority of women intend to and initiate breastfeeding, continuation of exclusive breastfeeding until an infant is six months of age is suboptimal and lower among women with complex socioeconomic and health needs. As the first Australian study to investigate associations between maternal, pregnancy and infant characteristics and reasons for early breastfeeding cessation, the findings show that women’s age and education level are associated with perceived insufficient milk supply. The most common reasons for breastfeeding cessation are potentially modifiable, through holistic care provision, highlighting that breastfeeding support responsive to women's personal priorities and situation is imperative during antenatal care and throughout the first six months of an infant’s life.

## Supplementary Information

**Additional file 1. **Associations between participants’ characteristics and reasons for breastfeeding cessation. Table depicting analysis of associations between maternal, pregnancy and infant characteristics and cited reasons for breastfeeding cessation.

## Data Availability

The datasets used and/or analysed during the current study are available from the corresponding author on reasonable request.

## Abbreviations

AMIHS: Aboriginal Maternal Infant Health Service
AUDIT-C: Alcohol Use Disorders Identification Test- Consumption
BMI: Body Mass Index
CATI: Computer Assisted Telephone Interview
CI: Confidence Interval
NSW: New South Wales
OR: Odds Ratio
REDCap: Research Electronic Data Capture
TAFE: Technical and Further Education
U.K.: United Kingdom
U.S: United States of America
