# Supplementary material for: Breastfeeding practices and associations with pregnancy, maternal and infant characteristics in Australia: a cross-sectional study
Source: Int Breastfeed J. 2023 Jan 19;18:8. doi: 10.1186/s13006-023-00545-5 (PMC9854140; doi:10.1186/s13006-023-00545-5)
Supplement: Supplementary file 1 — Additional file 1. Associations between participants’ characteristics and reasons for breastfeeding cessation. Table depicting analysis of associations between maternal, pregnancy and infant characteristics and cited reasons for breastfeeding cessation. [file 13006_2023_545_MOESM1_ESM.docx]

**Additional file 1.** Associations between participants’ characteristics and reasons for breastfeeding cessation (N=121).

| **Characteristic** | **Low milk supply (N=48)** | | | **Breastfeeding challenges (N=57)** | | | **Woman's preference (N=16)** | | | **Medical condition (N=23)** | | |
| --- | --- | --- | --- | --- | --- | --- | --- | --- | --- | --- | --- | --- |
|  | **n (%)** | **Odds ratio (95% CI)** | **p-value** | **n (%)** | **Odds ratio (95% CI)** | **p-value** | **n (%)** | **Odds ratio (95% CI)** | **p-value** | **n (%)** | **Odds ratio (95% CI)** | **p-value** |
| **Age of woman (years)** |  |  | .015^b^ |  |  | .053 |  |  | .25 |  |  | .55 |
| 18-25^a^ | 7 (20.6%) | 1.0 |  | 18 (52.9%) | 1.0 |  | 7 (20.6%) | 1.0 |  | 6 (17.7%) | 1.0 |  |
| 26-35 | 28 (43.1%) | 2.92 (1.11, 7.66) |  | 34 (52.3%) | 0.97 (0.42, 2.24) |  | 8 (12.3%) | 0.54 (0.18, 1.65) |  | 11 (16.9%) | 0.95 (0.32, 2.84) |  |
| 36+ | 13 (59.1%) | 5.57 (1.70, 18.29) |  | 5 (22.7%) | 0.26 (0.08, 0.87) |  | 1 (4.6%) | 0.18 (0.02, 1.61) |  | 6 (27.3%) | 1.75 (0.48, 6.34) |  |
| **Age of infant (months)** |  |  | .64 |  |  | .08 |  |  | .05 |  |  | .88 |
| 2 months^a^ | 9 (34.6%) | 1.0 |  | 17 (65.4%) | 1.0 |  | 0 (0%) | 1.0 |  | 5 (19.2%) | 1.0 |  |
| 3 months | 19 (45.2%) | 1.56 (0.57, 4.29) |  | 20 (47.6%) | 0.48 (0.18, 1.32) |  | 7 (16.7%) | 6.81 (1.26, ∞) |  | 7 (16.7%) | 0.84 (0.24, 2.99) |  |
| 4+ months | 20 (37.7%) | 1.14 (0.43, 3.05) |  | 20 (37.7%) | 0.32 (0.12, 0.86)^b^ |  | 9 (17.0%) | 7.09 (1.37, ∞) |  | 11 (20.8%) | 1.10 (0.34, 3.58) |  |
| **Aboriginal or Torres Strait Islander, or both** |  |  | .90 |  |  | .87 |  |  | .95 |  |  | .63 |
| Yes | 3 (37.5%) | 0.91 (0.21, 3.98) |  | 4 (50.0%) | 1.13 (0.27, 4.75) |  | 1 (12.5%) | 0.93 (0.11, 8.13) |  | 1 (12.5%) | 0.59 (0.07, 5.05) |  |
| No^a^ | 45 (39.8%) | 1.0 |  | 53 (46.9%) | 1.0 |  | 15 (13.3%) | 1.0 |  | 22 (19.5%) | 1.0 |  |
| **Marital status** |  |  | .40 |  |  | .94 |  |  | .67 |  |  | .77 |
| Never married | 5 (35.7%) | 0.88 (0.27, 2.80) |  | 6 (42.9%) | 0.83 (0.27, 2.55) |  | 3 (21.4%) | 1.88 (0.30, 8.52) |  | 2 (14.3%) | 0.65 (0.07, 3.29) |  |
| Separated or divorced | 3 (75.0%) | 4.72 (0.47, 46.9) |  | 2 (50.0%) | 1.10 (0.15, 8.12) |  | 0 (0%) | 1.35 (0.00, 8.38) |  | 0 (0%) | 0.76 (0.00, 4.62) |  |
| Married or de facto^a^ | 40 (38.8%) | 1.0 |  | 49 (47.6%) | 1.0 |  | 13 (12.6%) | 1.0 |  | 21 (20.4%) | 1.0 |  |
| **Education** |  |  | .031^b^ |  |  | .98 |  |  | .29 |  |  | .08 |
| Completed high school or less | 19 (43.2%) | 0.63 (0.26, 1.57) |  | 21 (47.7%) | 1.10 (0.44, 2.71) |  | 8 (18.2%) | 1.24 (0.37, 4.22) |  | 7 (15.9%) | 1.89 (0.45, 7.95) |  |
| Completed TAFE Certificate or Diploma | 11 (25.0%) | 0.28 (0.11, 0.73) |  | 21 (47.7%) | 1.10 (0.44, 2.71) |  | 3 (6.8%) | 0.41 (0.09, 1.85) |  | 13 (29.6%) | 4.19 (1.08, 16.21)^b^ |  |
| Completed University, CAE, Degree or higher^a^ | 18 (54.6%) | 1.0 |  | 15 (45.5%) | 1.0 |  | 5 (15.2%) | 1.0 |  | 3 (9.1%) | 1.0 |  |
| **Disadvantage** |  |  | .66 |  |  | .37 |  |  | .83 |  |  | .81 |
| Most disadvantaged | 27 (38.0%) | 0.85 (0.41, 1.77) |  | 31 (43.7%) | 0.72 (0.35, 1.48) |  | 9 (12.7%) | 0.89 (0.31, 2.58) |  | 14 (19.7%) | 1.12 (0.44, 2.83) |  |
| Least disadvantaged^a^ | 21 (42.0%) | 1.0 |  | 26 (52.0%) | 1.0 |  | 7 (14.0%) | 1.0 |  | 9 (18.0%) | 1.0 |  |
| **Pre-pregnancy BMI** | **(N=42)** |  | .38 | **(N=49)** |  | .39 | **(N=11)** |  | .06 | **(N=22)** |  | .32 |
| Underweight (<18.5kg/m^2^) | 1 (100%) | 1.46 (0.08, ∞) |  | 0 (0%) | 1.46 (0.00, 27.77) |  | 0 (0%) | 4.33 (0.00, 82.33) |  | 0 (0%) | 7.00 (0.00, 133.00) |  |
| Healthy weight^a^ (18.5-24.9 kg/m^2^) | 12 (38.7%) | 1.0 |  | 13 (41.9%) | 1.0 |  | 6 (19.4%) | 1.0 |  | 4 (12.9%) | 1.0 |  |
| Overweight (25.0-29.9 kg/m^2^) | 9 (31.0%) | 0.72 (0.21, 2.35) |  | 17 (58.6%) | 1.94 (0.63, 6.21) |  | 4 (13.8%) | 0.67 (0.12, 3.23) |  | 5 (17.2%) | 1.40 (0.27, 7.91) |  |
| Obese (≥30.0 kg/m^2^) | 20 (45.5%) | 1.31 (0.47, 3.75) |  | 19 (43.2%) | 1.05 (0.38, 2.97) |  | 1 (2.3%) | 0.10 (0.01, 0.85) |  | 13 (29.6%) | 2.79 (0.74, 13.18) |  |
| **Pregnancy** |  |  | .17 |  |  | .58 |  |  | .54 |  |  | .60 |
| First pregnancy | 17 (32.7%) | 0.60 (0.28, 1.26) |  | 23 (44.2%) | 0.82 (0.40, 1.68) |  | 8 (15.4%) | 1.39 (0.48, 3.98) |  | 11 (21.2%) | 1.27 (0.51, 3.17) |  |
| Subsequent pregnancy^a^ | 31 (44.9%) | 1.0 |  | 34 (49.3%) | 1.0 |  | 8 (11.6%) | 1.0 |  | 12 (17.4%) | 1.0 |  |
| **Type of birth** |  |  | .86 |  |  | .78 |  |  | .32 |  |  | .21 |
| Vaginal | 31 (40.3%) | 1.07 (0.50, 2.29) |  | 37 (48.1%) | 1.11 (0.53, 2.33) |  | 12 (15.6%) | 1.85 (0.56, 6.12) |  | 12 (15.6%) | 0.55 (0.22, 1.39) |  |
| Caesarean^a^ | 17 (38.6%) | 1.0 |  | 20 (45.5%) | 1.0 |  | 4 (9.1%) | 1.0 |  | 11 (25.0%) | 1.0 |  |
| **Pre-eclampsia** |  |  | .99 |  |  | .75 |  |  | 1.00 |  |  | .24 |
| No | 46 (39.7%) | 0.99 (0.16, 6.13) |  | 55 (47.4%) | 1.35 (0.22, 8.40) |  | 16 (13.8%) | 1.05 (0.18, ∞) |  | 21 (18.1%) | 0.33 (0.05, 2.11) |  |
| Yes^a^ | 2 (40.0%) | 1.0 |  | 2 (40.0%) | 1.0 |  | 0 (0%) | 1.0 |  | 2 (40.0%) | 1.0 |  |
| **Gestational Diabetes** |  |  | .30 |  |  | .26 |  |  | .67 |  |  | .12 |
| No/Don’t know^a^ | 42 (38.2%) | 1.0 |  | 50 (45.5%) | 1.0 |  | 15 (13.6%) | 1.0 |  | 23 (20.9%) | 1.0 |  |
| Yes | 6 (54.6%) | 1.94 (0.56, 6.76) |  | 7 (63.6%) | 2.10 (0.58, 7.59) |  | 1 (9.1%) | 0.63 (0.08, 5.31) |  | 0 (0%) | 0.25 (0.00, 1.28) |  |
| **Pregnancy risk level** |  |  | .94 | **(N=56)** |  | .07 | **(N=15)** |  | .24 |  |  | .16 |
| Low risk (midwives) | 29 (39.7%) | 0.97 (0.46, 2.05) |  | 39 (53.4%) | 2.02 (0.95, 4.29) |  | 7 (9.6%) | 0.52 (0.17, 1.54) |  | 11 (15.1%) | 0.52 (0.21, 1.29) |  |
| High risk (medical clinic, vulnerable, medical needs)^a^ | 19 (40.4%) | 1.0 |  | 17 (36.2%) | 1.0 |  | 8 (17.0%) | 1.0 |  | 12 (25.5%) | 1.0 |  |
| **Smoked tobacco at time of survey** |  |  | .94 |  |  | .08 |  |  | .64 |  |  | .71 |
| No^a^ | 41 (40.2%) | 1.0 |  | 52 (50.5%) | 1.0 |  | 13 (12.6%) | 1.0 |  | 19 (18.5%) | 1.0 |  |
| Yes | 7 (38.9%) | 0.96 (0.34, 2.39) |  | 5 (26.3%) | 0.34 (0.12, 1.02) |  | 3 (16.7%) | 1.38 (0.35, 5.45) |  | 4 (22.2%) | 1.26 (0.37, 4.27) |  |
| **Alcohol consumption at time of survey** |  |  | .95 |  |  | .22 |  |  | .72 |  |  | .36 |
| No risk of harm (score = 0)^a^ | 14 (35.9%) | 1.0 |  | 20 (51.3%) | 1.0 |  | 6 (15.4%) | 1.0 |  | 9 (23.1%) | 1.0 |  |
| Low risk of harm (score = 1-2) | 21 (42.0%) | 1.29 (0.55, 3.06) |  | 26 (52.0%) | 1.03 (0.45, 2.38) |  | 7 (14.0%) | 0.09 (0.27, 2.92) |  | 8 (16.0%) | 0.63 (0.22, 1.84) |  |
| Medium risk of harm (score = 3-4) | 8 (40.0%) | 1.19 (0.39, 3.61) |  | 9 (45.0%) | 0.78 (0.26, 2.29) |  | 1 (5.0%) | 0.29 (0.03, 2.59) |  | 2 (10.0%) | 0.37 (0.07, 1.91) |  |
| High risk of harm (score = 5+) | 5 (41.7%) | 1.28 (0.34, 4.78) |  | 2 (16.7%) | 0.19 (0.04, 0.98) |  | 2 (16.7%) | 1.10 (0.19, 6.33) |  | 4 (33.3%) | 1.67 (0.41, 6.84) |  |

**^a^** Reference value for Odds Ratio

**^b^** Statistically significant at (α=0.05)
